# Supplementary material for: Curated and harmonised transcriptomics datasets of interstitial lung diseases
Source: Data Brief. 2025 Oct 14;63:112139. doi: 10.1016/j.dib.2025.112139 (PMC12581653; doi:10.1016/j.dib.2025.112139)
Supplement: Supplementary file 1 [file mmc1.zip › Supplementary_material/RNA-seq/fastQC reports/GSE184316/TRIMMED_SRR15926761_fastqc.html]

TRIMMED\_SRR15926761.fastq.gz FastQC Report 

FastQC Report

Sat 3 Dec 2022  
TRIMMED\_SRR15926761.fastq.gz

## Summary

- Basic Statistics
- Per base sequence quality
- Per sequence quality scores
- Per base sequence content
- Per sequence GC content
- Per base N content
- Sequence Length Distribution
- Sequence Duplication Levels
- Overrepresented sequences
- Adapter Content

## Basic Statistics

| Measure | Value |
| --- | --- |
| Filename | TRIMMED\_SRR15926761.fastq.gz |
| File type | Conventional base calls |
| Encoding | Sanger / Illumina 1.9 |
| Total Sequences | 7152842 |
| Sequences flagged as poor quality | 0 |
| Sequence length | 35-372 |
| %GC | 50 |

## Per base sequence quality

## Per sequence quality scores

## Per base sequence content

## Per sequence GC content

## Per base N content

## Sequence Length Distribution

## Sequence Duplication Levels

## Overrepresented sequences

| Sequence | Count | Percentage | Possible Source |
| --- | --- | --- | --- |
| CAGTGGGGCCACGAGCTGAGTGCGTCCTGTCACTCCACTCCCATGTCCCT | 67847 | 0.9485320659955861 | No Hit |
| TTACAAAAGCAGAATAAAAGCGAAAAGAAATGAAAATGTTACACTACATT | 52189 | 0.7296260703088366 | No Hit |
| CACCTCCCCGAAGCTCAGGGAGAGCCCTGTTAGGGCCGCCTCTGGCCCTA | 50492 | 0.7059012347819231 | No Hit |
| CAATCCACTTGATCCCAACTCATCTCTCATTTATTTCGGCTTCTTTTATT | 48788 | 0.682078536056018 | No Hit |
| TACCACTGCCACCTTCTCCATCGGCTCCACTGGCCTCGTGGTGTATGACT | 37967 | 0.5307960108723219 | No Hit |
| AGACATCAAATCCTGCAGACAAGGGGAGCCCTCAGTCTGCAGGGCTCCAT | 37282 | 0.521219397828164 | No Hit |
| GGCATCAAAAGTGATGGACTGCCCATTGCTGGAGAAGACCTTCTCTCCTA | 35116 | 0.49093772796882695 | No Hit |
| CTTCATGATGTAGCAGCAGGTGCCAGGGGCTGGCTTGTAGGCGATCAGCA | 34614 | 0.4839195385554441 | No Hit |
| AGGCTACACACTGAGGACTCTGTTCCTCCCCTTTCCGCCTAGGGGAAAGT | 32249 | 0.45085575775335174 | No Hit |
| TTCAGCCCTGATCAAGACATGAGGGAGGCAGGGGCTCAGCTGAAGAAGCT | 29211 | 0.40838312939108684 | No Hit |
| TGATATGGTTCCTGGCAAGCCCATGTGTGTTGAGAGCTTCTCAGACTATC | 26649 | 0.3725651985602366 | No Hit |
| TGATGACACCCACCGCAACTGTCTGTCTCATATCACGAACAGCAAAGCGA | 25555 | 0.35727057860358163 | No Hit |
| GGGAAGCGGGGAATGTCTACGTGCGTATGCACGTGGCACTCTCTGCCCGA | 24900 | 0.3481133792693869 | No Hit |
| GGCAATGAGCGGTTCCGCTGCCCTGAGGCACTCTTCCAGCCTTCCTTCCT | 18927 | 0.2646081096157304 | No Hit |
| TTTTCCATGAGCTTAATGATGCTTTCTCTGGGCTTTTGGGGGAGGGTGTC | 17694 | 0.24737020613624627 | No Hit |
| ACAAGAAGTACATCAAAGATTACATGAAATCAATCAAAGGGAAACTTGAA | 16724 | 0.2338091628474388 | No Hit |
| GTCCACGTCACACTTCATGATGGAGTTGAAGGTAGTTTCGTGGATGCCAC | 16645 | 0.23270470674453597 | No Hit |
| GAAACCCAGACACATAGCAATTCAGGAAATTTGACTTTCCATTCTCTGCT | 16635 | 0.23256490217454825 | No Hit |
| CTCTCTTTCTGGCCTGGAGGCTATCCAGCGTACTCCAAAGATTCAGGTTT | 15305 | 0.2139708943661834 | No Hit |
| GTTCTGCAGCCCCTGTCATAAAAGGTTTTACTCTTTCTGGTCTCTGTTCT | 12794 | 0.17886596684227055 | No Hit |
| CCCTGGCTGCTACAGGAGAATAGCAGACAGGTATAGTTAAGAAACAACTT | 11209 | 0.1567069424992192 | No Hit |
| CAATGGGGTACTTCAGGGTCAGGATGCCACGCTTGCTCTGGGCCTCGTCG | 10991 | 0.15365920287348722 | No Hit |
| CAGCATGTTCCCTCTCCTCATGAGATTGGTGAAGAAAGTATTTGGCAAAG | 10828 | 0.1513803883826876 | No Hit |
| ACGTTTACCTGTCCATGTCTTACTACTTTGACCGCGATGATGTGGCTTTG | 10672 | 0.1491994370908794 | No Hit |
| AGCTGCTAAGTGCTGTGTTGTCGTTCCCCCTGCTTAAAATAAAGTTGTTT | 10549 | 0.1474798408800306 | No Hit |
| TTTTTCCATGAGCTTAATGATGCTTTCTCTGGGCTTTTGGGGGAGGGTGT | 9080 | 0.12694254954883666 | No Hit |
| ATCAAAGAAGAACATGTGATCATCCAGGCCGAGTTCTATCTGAATCCTGA | 9037 | 0.12634138989788954 | No Hit |
| GGCATCAAAAGTGATGGACTGCCCATTGCTAGAGAAGACCTTCTCTCCTA | 8656 | 0.12101483578135797 | No Hit |
| GTCCACGTCACACTTCATGATGGAGTTGAAGGTGGTCTCGTGGATGCCGC | 8556 | 0.11961679008148089 | No Hit |
| GGCAATGAGCGGTTCCGGTGTCCGGAGGCGCTGTTCCAGCCTTCCTTCCT | 8530 | 0.11925329819951287 | No Hit |
| GTCTCAACATCTGTGTTGGGGAGAGTGGAGACAGACTGACGCGAGCAGCC | 7864 | 0.10994231383833167 | No Hit |
| CGAGCCACAGCTCTGCCAGTACCCCAAGACTCAGCACTAGTCTGATGACC | 7813 | 0.10922931053139438 | No Hit |
| CAGCAGTTTACACCTGCTAGGGTGTTCAAAGGTCAGTGCTATAGAAATTC | 7784 | 0.10882387727843004 | No Hit |
| CATATGGCTCTCCCAGAGGCAGAAGGACAATGGCTGTTTCAGGAGCTCTG | 7571 | 0.10584603993769189 | No Hit |
| CTGACAGTGTATCTAGCTTTGGAAAACACAGGGGTCTGCCCTGTGAGCTG | 7345 | 0.10268645665596976 | No Hit |
| CGATCAGCACGGGCTCGTACTCTGCCACAAACTGATCACACTGCTTCTGG | 7244 | 0.10127443049909393 | No Hit |
| AACAAGCACAAAGCCAAGAAAACCAACGATGCCAGATACTGAATTTCTAT | 7155 | 0.10003016982620333 | No Hit |

## Adapter Content

Produced by FastQC (version 0.11.7)
